# Efficacy of Polymer-Based Nanocarriers for Co-Delivery of Curcumin and Selected Anticancer Drugs

**DOI:** 10.3390/nano10081556

**Published:** 2020-08-08

**Authors:** Sibusiso Alven, Blessing Atim Aderibigbe

**Affiliations:** Department of Chemistry, University of Fort Hare, Alice Eastern Cape 5700, South Africa; 201214199@ufh.ac.za

**Keywords:** cancer, anticancer drugs, curcumin, combination therapy, polymer-based nanocarriers

## Abstract

Cancer remains a heavy health burden resulting in a high rate of mortality around the world. The presently used anticancer drugs suffer from several shortcomings, such as drug toxicity, poor biodegradability and bioavailability, and poor water solubility and drug resistance. Cancer is treated effectively by combination therapy whereby two or more anticancer drugs are employed. Most of the combination chemotherapies result in a synergistic effect and overcome drug resistance. Furthermore, the design of polymer-based nanocarriers for combination therapy has been reported by several researchers to result in promising therapeutic outcomes in cancer treatment. Curcumin exhibits good anticancer activity but its poor bioavailability has resulted in its incorporation into several polymer-based nanocarriers resulting in good biological outcomes. Furthermore, the incorporation of curcumin together with other anticancer drugs have been reported to result in excellent therapeutic outcomes in vivo and in vitro. Due to the potential of polymer-based nanocarriers, this review article will be focused on the design of polymer-based nanocarriers loaded with curcumin together with other anticancer drugs.

## 1. Introduction

Cancer is explained as a severe metabolic disease that causes a high rate around the world although there are various available interventions [1,2]. There were about 14 million cases of this disease diagnosed in 2012 with approximately 8.2 million cancer-associated deaths reported [3]. The World Health Organisation (WHO) predicted that in 20 years, cases of cancer will rise by 70% [3]. Internal factors that contribute to cancer are genetic mutation, hormones and immune condition, and external factors are pollution, smoking, radiation, and infectious organisms [1]. There are several types of cancer (e.g., lung, colon cancer, skin, ovarian, bone, prostate, liver, breast, colorectal cancer, etc.). However, the most common types are lung, breast, and colorectal cancer [4]. Various approaches are utilized in cancer treatment including surgery, immunotherapy, radiotherapy, hormonal therapy, and chemotherapy (anticancer drugs) [5,6].

Chemotherapy is the most utilized method and has been known as one of the most effective approaches for the treatment of cancer in clinical practice [5]. Nevertheless, the use of chemotherapeutic agents is hampered by the three major limitations such as poor water solubility, poor bioavailability, and non-specific target with adverse effects on healthy body cells [7]. The second limitation results in non-uniform biodistribution thereby causing poor localization of chemotherapeutic agents at the tumor environment. The aforementioned limitation usually results in drug toxicity [8,9]. Targeting one pathway with conventional chemotherapy is not an effective approach for the treatment of cancer, and it usually results in the development of multidrug resistance [10].

Combination chemotherapy is a promising approach that is utilized to combat the aforementioned limitations associated with most anticancer drugs [11]. Combination chemotherapy involves the concurrent administration of two or more anticancer agents with different modes of action so as overcome multidrug resistance [11]. Curcumin, a therapeutic agent with several biological actions including anti-androgenic, anti-inflammatory, anti-oxidant, wound healing, anti-angiogenic, and anticancer efficacy [12]. Curcumin has been used in combination with other bioactive agents with good therapeutic outcomes in vitro and in vivo. Furthermore, polymer-based nanocarriers such as nanoparticles [13,14,15,16,17], nanoliposomes [18,19,20,21,22], nanocapsules [23,24,25,26], nanomicelles [27], polymer-drug conjugates [28,29], hydrogels [30], and dendrimers [31,32,33,34] have been reported for the combination of curcumin and other therapeutic agent(s) for the treatment of cancer [35]. There are several advantages of employing polymer-based nanocarriers in the treatment of cancer such as reduced drug toxicity, improved cellular uptake and internalization of anticancer drugs, increased drug solubility, improved drug bioavailability and biodegradability, controlled and sustained drug release kinetics, and enhanced patient compliance [36,37]. This article will focus on interesting therapeutic outcomes of polymeric nanocarriers co-loaded with curcumin for the treatment of cancer in vitro and in vivo.

## 2. Curcumin in Cancer Therapy

**Figure 1:**
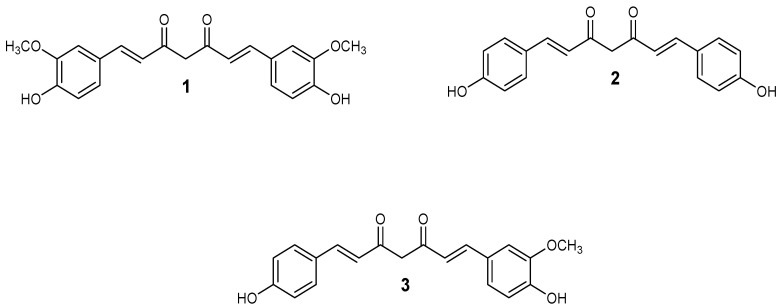
Chemical structure of curcumin, bisdemethoxycurcumin, and demethoxycurcumin.

Numerous chemotherapeutics with various mechanisms of action have been extracted from plant sources, such as *Catharanthus roseus*, *Taxus brevifolia, Betula alba*, *Curcuma longa*, *Erythroxylum previllei*, and *Cephalotaxus* species, and *Curcuma longa L*, (where curcumin is isolated) [38]. Curcumin is generally a yellow powder, and it is an effective constituent of the Indian spice turmeric, which is extracted from dried *Curcuma longa* plant [39,40]. Turmeric has three major components (Figure 1): curcumin **1**, bisdemethoxycurcumin **2** and demethoxycurcumin **3**, and curcumin is the most abundant and active component [41]. Curcumin and its analogues have been reported to possess anticancer activity on several cancer cell lines, such as lung, colorectal, ovarian, pancreatic, breast carcinoma, oral, and melanoma cells. However, there are some pharmacological limitations associated with the use of curcumin such as low bioavailability and poor water solubility [42].

The mode of action of curcumin against cancer is via apoptosis and proliferation inhibition, anti-angiogenesis by preventing the development of blood supply needed by the cancer cell for growth, hindering the synthesis of proteins needed for cancer formation and destroying a variety of cellular signaling pathways, etc. [12]. Curcumin hinders the nuclear factor kappa B (NF-*k*B) and Signal Transducer and Activator of Transcription (STATS) signaling pathways that perform vital roles in the formation and development of cancer. Curcumin also inhibits Sp-1 and its gene expression to avoid cancer development, invasion, and migration. Recent studies have indicated that curcumin can act by terminating Sp-1 stimulation and its downstream genes, such as EPHB2, SEPP1, HDAC4, calmodulin, and ADEM10, depending on the concentration of curcumin, in colorectal and bladder cancer cells. It also disrupts the activity of DNA binding of Sp-1 in non-small cell lung carcinoma cells. Furthermore, a study demonstrated that autophagy and Endoplasmic Reticulum (ER) stress may play a significant role in the process of apoptosis in an ovarian cancer cell line, which is stimulated by the curcumin analogue B19. The inhibition of autophagy enhances curcumin analogues capability to induce apoptosis by stimulating serious ER stress [12]. 

Clinical studies of curcumin administered in combination with selected anticancer drugs resulting in good therapeutic outcomes have been reported (Table 1). Howells et al. reported a phase IIa open-labelled randomized controlled trial in which there was a comparison of 2 g of curcumin plus folinic acid/fluorouracil/oxaliplatin with folinic acid/5-fluorouracil/oxaliplatin chemotherapy. Twenty-eight patients with metastatic colorectal cancer were administered either 2 g of curcumin plus folinic acid/fluorouracil/oxaliplatin or folinic acid/5-fluorouracil/oxaliplatin. Curcumin did alter CXCL1 over time significantly with (*p* = 0.712). It was found to be safe and tolerable for chemotherapy in patients with metastatic colorectal cancer when used in combination with folinic acid/5-fluorouracil/oxaliplatin [43]. Kanai et al. reported the efficacy of combining curcumin with gemcitabine-based chemotherapy in patients with pancreatic cancer. Twenty-one patients were enrolled and administered orally 8 g of curcumin daily in combination with gemcitabine. The median survival time after the administration of curcumin was 161 days with a 1-year survival rate. The plasma curcumin levels in five patients were in the range of 29 to 412 ng/mL. The combination was found to be safe for patients with pancreatic cancer [44]. Bayet-Robert et al. reported the efficacy of combining docetaxel and curcumin in patients with metastatic breast cancer [45]. Patients with advanced or metastatic breast cancer were administered docetaxel (100 mg/m^2^) in a 1 h intravenous infusion every three weeks on day 1 for six cycles. A 500 mg/d dose of curcumin was administered orally for seven consecutive days by cycle (from d−4 to d+ 2) and escalated until dose-limiting toxicity occurred. A recommended dose of 6,000 mg/d of curcumin for seven consecutive days every three weeks when used in combination with a standard dose of docetaxel was reported. Ghalaut et al. reported the efficacy of combining imatinib with curcumin in patients with chronic myeloid leukemia. Fifty patients with chronic myeloid leukemia were enrolled in this trial. One group were administered imatinib alone and the second group was administered a combination of turmeric powder with imatinib for a period of six weeks. The levels of nitric oxide were significantly decreased in the second group revealing that curcumin acts as an adjuvant when combined with imatinib, which is useful in the treatment of chronic myeloid leukemia patients [46].

Mahammedi et al. reported the findings of a clinical trial that involved the combination of docetaxel with curcumin in patients with chemotherapy-naive metastatic castration-resistant prostate cancer. Thirty patients were administered docetaxel/prednisone in standard conditions for six cycles in combination with curcumin (6,000 mg/day) (day −4 to day +2 of docetaxel). The combination did not result in any adverse effect and it was well tolerated [47]. Pastorelli et al. reported a study which addressed the safety and efficacy of combining curcumin with gemcitabine in patients with pancreatic cancer. The patients received a combination of gemcitabine and Meriva^®^, patented curcumin complexed with phospholipids. Fifty-two patients were enrolled in the study. The median progression free survival and overall survival were 8.4 and 10.2 months, respectively. Increased sCD40L levels were observed after one cycle of chemotherapy resulting from a lowered response to the therapy. The combination was safe and can translate into a good response rate in first line therapy of advanced pancreatic cancer [48]. 

Chen et al. investigated the efficacy of combining MB-6 composed of fermented soybean extract, green tea extract, antrodia camphorata mycelia, spirulina, grape seed extract, and curcumin extract with leucovorin/5-fluorouracil/oxaliplatin in 72 patients with metastatic colorectal cancer. They were administered leucovorin, 5-fluorouracil, and oxaliplatin in combination with either MB-6 or placebo for 16 weeks. After 77 weeks of treatment, the patients in the MB-6 group displayed a significantly lower rate of disease progression when compared to patients in the placebo group. The placebo group had a higher incidence of adverse events when compared to the MB-6 group [49]. Panahi et al. reported a randomized double-blind placebo-controlled trial that investigated the efficacy of curcuminoids as adjuvant therapy in combination with 5-fluorouracil–based chemotherapy in cancer patients. Eighty patients with solid tumors who were under standard chemotherapy regimens were administered curcuminoid (180 mg/day; *n* = 40 patients) or matched placebo (*n* = 40 patients) for a period of eight weeks. The efficacy of the combination was measured by the health-related quality of life score and the serum levels of a panel of mediators implicated in systemic inflammation such as interleukins 6 (IL-6) and 8 (IL-8), etc. The combination resulted in a significant improvement in health-related quality of life when compared to the placebo (*p*  < 0.001). The magnitude of reductions in the mediators implicated in systemic inflammation was significant in the curcuminoids when compared to placebo group, revealing a suppression of systemic inflammation in patients with solid tumors [50]. Ryan et al. conducted a randomized, double-blind, placebo-controlled clinical trial to evaluate the potential of curcumin in the reduction of the severity of radiation dermatitis in thirty breast cancer patients. The patients were administered 2 g of curcumin or placebo orally, three times per day (a total of 6.0 g per day). The oral curcumin of 6 g daily during radiotherapy resulted in the reduced severity of radiation dermatitis in breast cancer patients [51]. Furthermore, Esfahani et al. reported a phase 1 open prospective study to investigate the safety of administering a combination of curcumin formulation (CURCUViva^TM^) at 80 mg/1 capsule daily in combination with tyrosine kinase inhibitors (TKIs), gefitinib, and erlotinib in patients with advanced non-small cell lung cancer. Fifty-five patients were enrolled with an 80% completion rate and 82% adherence levels. The short-term use of curcumin in combination with EGFR-positive patients treated with TKIs was safe [52]. Kanai et al. developed a curcumin formulation, Theracurmin^®^, and evaluated its safety with repetitive administration in pancreatic or biliary tract cancer patients. It was also assessed when combined with standard gemcitabine-based chemotherapy. Theracurmin^®^ containing 200 mg of curcumin was used as the starting dose, and the dose was safely increased to 400 mg of curcumin. Theracurmin^®^ was also administered orally in combination with standard gemcitabine-based chemotherapy. No adverse effects were observed in the patients. The repetitive administration of a high dosage of curcumin did not increase the incidence of adverse effects in cancer patients receiving curcumin together with the gemcitabine-based chemotherapy [53].

There are reports from clinical trials which have been carried out to investigate the effects of curcumin in cancer patients showing that it improved tumor markers and provided symptomatic relief. It has been investigated on different types of cancer such as the brain, multiple myeloma, head, neck, colon, breast, colorectal, etc. Kim et al. reported the ability of curcumin to inhibit IkB kinase b (IKKb) activity in the saliva of head and neck squamous cell carcinoma cancer patients, thereby suppressing the expression of proinflammatory cytokines [54]. Ide et al. reported the potential of combining curcumin with isoflavones to reduce the level of prostate-specific antigen (PSA), which indicates the presence of inflammation in prostate cancer. Eighty-five participants were given a supplement containing isoflavones and curcumin or placebo daily. PSA level was decreased in patients who were treated with a combination of isoflavones and curcumin revealing that isoflavones and curcumin can influence the serum PSA levels via anti-androgen effects [55]. He et al. reported the inhibitory effect of curcumin in colorectal cancer patients. The administration of curcumin increased body weight and reduced serum TNF-alpha levels, increased apoptotic tumor cells, and enhanced the expression of the p53 molecule in tumor tissue. Curcumin improved the general health of patients with colorectal cancer by increasing p53 molecule expression in tumor cells and accelerating tumor cell apoptosis [56]. Clinical studies of curcumin have also been reported. Curcumin have been administered via intravaginal routes via cream and capsules in patients with cervical human papilloma virus. The vaginal cream known as Basant™ is composed of curcumin extract, amla, aloe vera, and reetha. The vaginal capsules are composed of 500 mg of curcumin in each capsule. A clinical trial by Basu et al. in which 287 human papillomavirus (HPV) positive women without high grade cervical neoplasia were treated with vaginal placebo cream, vaginal Basant cream, curcumin vaginal capsules, and placebo vaginal capsules, respectively. The formulation was administered once a day for 30 consecutive days except during menstruation. After seven days of the last application, the HPV test was repeated. The HPV clearance rate in Basant cream was significant with (87.7%) when compared to the combined placebo arms which was (73.3%). No serious adverse events were observed [57]. Ora-Curcumin E formulation of curcumin has also been licensed to Academic Technology Ventures Inc. The formulation dissolves in the acidic pH of the stomach. This formulation improved the solubility of curcumin significantly with good site-specific targeting ability [58].

## 3. Nanocarriers for Combination Chemotherapy 

Among the strategies that are used to overcome the limitations associated with anticancer drugs, combination chemotherapy is one of the most effective strategies [59]. Recently, combination chemotherapy has been greatly evaluated and the clinical practice outcomes have revealed synergistic cytotoxic effects which were more potent when compared to a single anticancer drug with reduced drug toxicity [60]. The synergistic effect of therapeutic agents when combined together using nanocarriers results from the presence of the active molar ratios of the combined drugs at the tumor environments, which is difficult to attain using the conventional administration procedures due to factors such as different pharmacokinetics properties, injection schedules, non-uniform distribution, and metabolism [60]. 

Nanocarriers are used for the co-delivery of selected chemotherapeutics to the tumor environment at a controlled or sustained drug release mechanism [61,62]. The pharmacokinetic characteristics of these nanocarriers can be modified with biodistribution of anticancer drugs. Nanocarriers also enhance the biological activity of the incorporated/encapsulated chemotherapeutic agents, reduce drug toxicity, and increase drug stability, prolong the plasma circulation period of the incorporated/encapsulated drug and enhance the drug accumulation and internalization at the tumor sites [63]. Furthermore, the development of good therapeutic activity can be accomplished by designing stimuli-responsive nanocarriers with target-activated moieties. Nanocarriers designed for co-delivery of drugs possess the potential of encapsulating hydrophilic and hydrophobic therapeutics [60].

**Figure 2:**
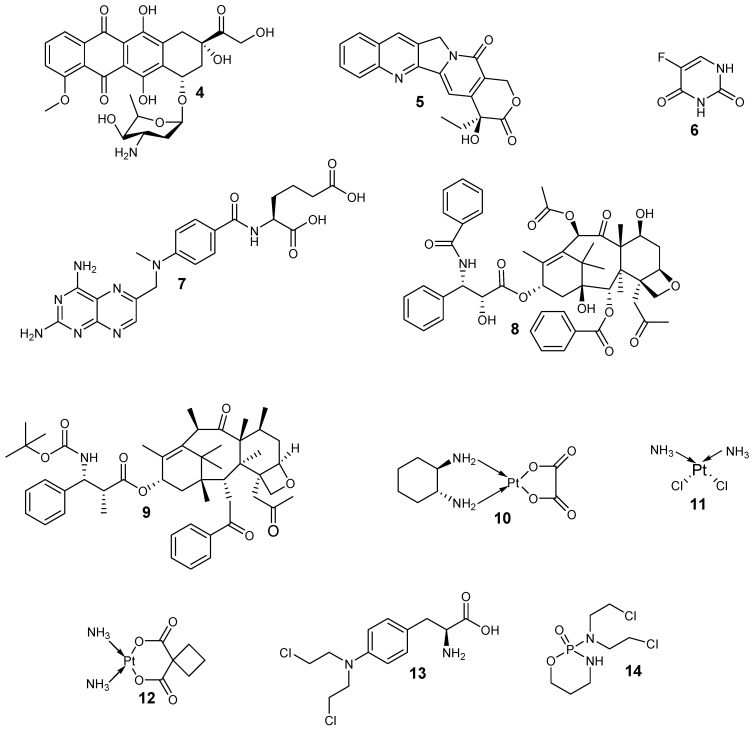
Chemical structure of anticancer drugs.

Although much development has been achieved with nanocarriers for the co-delivery of anticancer agents, some issues need to be considered in the preparation of ideal nanocarriers such as those related to the entrapment of therapeutic agents with a diversity of physicochemical properties and solubilities, increasing drug accumulation within the tumor site and controlling their sequential drug release. Several nanocarriers have been evaluated for the co-delivery of chemotherapeutics such as dendrimers, liposomes, polymeric nanoparticles, and lipid nanoparticles. The polymers that are used to prepare the nanocarriers are categorized into six classes: thermo-sensitive polymers, block co-polymer conjugates, pH-Sensitive polymers, MMP-sensitive, redox-sensitive polymers, and magnetic-responsive [44]. The combination of anticancer drugs and curcumin have been designed using nanocarriers (Figure 2). Examples anticancer drugs (Figure 2) that have been combined with curcumin are doxorubicin **4**, camptothecin **5**, 5-fluorouracil **6**, methotrexate **7**, paclitaxel **8**, docetaxel **9**, oxaliplatin **10**, cisplatin **11**, carboplatin **12**, melphalan **13** and cyclophosphamide **14.**

## 4. Polymer-Based Nanocarriers for Co-Delivery of Curcumin with Anticancer Drugs

### 4.1. Polymeric Nanoparticles

Nanoparticles are nanocarriers that are biocompatible solid particles with a size range of 1–1000 nm [64]. These nanocarriers are usually synthesized from biopolymers, semi-synthetic, and synthetic polymers in which the therapeutic agents are encapsulated or incorporated. There are several biodegradable polymers that are frequently employed for the formulation of polymeric nanoparticles including poly (D,L-lactic-co-glycolic)(PLGA), poly(D,L-lactic acid)(PLA), polyalkylcyanoacrylates) etc. [65]. Polymeric nanoparticles, particularly stimuli-sensitive polymeric nanoparticles, display a high drug loading capacity and a controlled drug release profile with improved in vivo stability for the co-delivery of various categories of anticancer agents [66]. Furthermore, other advantages of nanoparticles include reduced drug toxicity, improved aqueous solubility, and improved therapeutic activity of the drugs [67]. Polymeric nanoparticles have been designed for the delivery of curcumin and in combination with selected anticancer agents (Table 2).

Zhang et al. formulated pH-sensitive, polymer-based nanoparticles for the co-delivery of curcumin, an anti-angiogenic drug, and doxorubicin, a pro-apoptotic drug using amphiphilic poly(β-amino ester) copolymer [68]. The physicochemical characterization of the co-loaded nanoparticles showed high drug encapsulation efficiency, low polydispersity with a particle size of 100 nm, and improved drug release at acidic conditions of cancer cells. The cellular internalization studies revealed enhanced internalization of drugs delivered from nanoparticles in umbilical vein endothelial cancer cell lines (HUVECs) and human liver cancer cell lines (SMMC 7721) when compared to the free drugs. The cytotoxicity of the co-loaded polymeric nanoparticles against SMMC 7721 was evaluated using MTT assay. Free doxorubicin, doxorubicin combined with curcumin, and co-loaded nanoparticles demonstrated cytotoxicity in a concentration-dependent way. The high cytotoxicity of the combined drugs indicated that the co-encapsulation of doxorubicin and curcumin in polymeric nanoparticles significantly enhanced their capability to destroy SMMC 7721 cancer cells in vitro with an improved cellular uptake. Furthermore, the apoptosis rates of curcumin, doxorubicin, doxorubicin combined with curcumin, and co-loaded nanoparticles were 14.4%, 26.4%, 38.3%, and 76.2%, respectively [68]. In vivo studies showed that the body weights of mice treated with co-encapsulation of doxorubicin and curcumin in polymeric nanoparticles showed no significant loss when compared to the control group revealing the non-systemic toxicity of the formulation. The higher tumor targeting capacity of the formulation reduced the uptake of the drug in delicate tissues such as the heart, etc.

Yan and co-workers synthesized polymeric nanoparticles and lipid-polymer hybrid nanoparticles for the combination of curcumin and docetaxel using PLGA as a polymer, to combat prostate cancer [69]. The particle size analysis of these co-encapsulated nanoparticles showed a particle size of 169.6 nm and a positive zeta potential of 35.7 mV. Curcumin and docetaxel encapsulation efficiency of co-loaded hybrid nanoparticles was 82% and 89%, respectively. Curcumin and docetaxel encapsulation efficiency of co-loaded plain polymeric nanoparticles was 73% and 80%, respectively. The drug release evaluation in vitro was sustained for docetaxel from the lipid-polymer hybrid nanoparticles when compared to the polymeric nanoparticles. These lipid-polymer nanoparticles exhibited the highest cytotoxicity in vitro, and the synergistic effect of combining curcumin and docetaxel was significant in human prostate carcinoma cells (PC3 cells). In vivo studies using mice-bearing PC3 tumor xenografts showed that the hybrid nanoparticles inhibited tumor growth without causing any severe side effects. The structure of the nanoparticles played a key role in delaying the drug release thereby increasing the drug accumulation in the tumor tissues. Furthermore, the shell of the nanoparticles improved the fusion of the nanocarriers to the cell membrane resulting in a high drug uptake into the tumor cells [69].

Li and co-workers developed and compared lipid-polymeric hybrid nanoparticles and polymeric nanoparticles for the combination of curcumin and cisplatin using PLGA as a polymer [70]. The particle size diameter of the co-loaded hybrid nanoparticles and plain co-loaded nanoparticles were 163.4 and 118.5 nm, respectively. The surface charge of the hybrid nanoparticles was lower when compared to polymeric co-loaded nanoparticles. The drug encapsulation efficiency of cisplatin in the co-loaded hybrid nanoparticles and co-loaded polymeric nanoparticles was 88.7% and 83.3%, respectively. The encapsulation efficiency of curcumin was above 80% in both co-loaded hybrid nanoparticles and co-loaded polymeric nanoparticles. The in vitro release profiles of the loaded drugs were sustained for two days. Curcumin and cisplatin released from lipid-polymer hybrid nanoparticles was slower when compared to polymer nanoparticles. The in vivo anti-tumor activity studies on a cervical cancer-infected BALB/c mice model showed that the tumors treated with co-loaded hybrid nanoparticles displayed significant anti-tumor effect when compared to those administered with the co-loaded polymeric nanoparticles, single drug-loaded hybrid nanoparticles, and free drugs. The loading of the drug into the nanoparticles enhanced drug accumulation in the tumor tissue when compared to the free drugs. Their nano-size promoted their cellular uptake via enhanced permeability and retention (EPR) effects. The significant anti-tumor activity of the lipid-polymeric hybrid nanoparticles is attributed to their selective delivery of the drugs to the cancer site. The structure of lipid polymeric hybrid nanoparticles also contributes to delayed drug release, enhanced drug circulation time, and increased drug accumulation in tumor tissues. It promoted the fusion of the carriers to the cells, resulting in targeted drug delivery into the tumor cells [70].

Lin et al. formulated and characterized poly (ethylene glycol) (PEG)-lipid bilayer coated mesoporous silica nanoparticles for the co-delivery of curcumin and paclitaxel [71]. The drug encapsulation efficiency of curcumin and paclitaxel in the nanoparticles was 77.48% and 30.70%, respectively. The morphology of the co-loaded nanoparticles was spherical with uniform dispersion. The cytotoxic activity of the nanoparticles against breast cancer cells in vitro using CCK8 assay was highest at each single time point (48, 60 and 72 h) when compared to free curcumin-paclitaxel mixture solution. Furthermore, in vitro drug release analysis revealed controlled and sustained drug release from the PEGylated lipid bilayer coated silica nanocarriers [71].

Chen and co-workers synthesized mPEG-PLGA nanoparticles via the nano-precipitation method for dual delivery of curcumin and cisplatin, using phenylboronate ester and disulphide bond as linkers [72]. The particle size analysis of polymeric nanoparticles using dynamic light scattering (DLS) displayed average particle diameters of 154.87 ± 10.32 nm, while the polymer index (PI) and zeta potential were 0.115 ± 0.012 and 20.8 ± 3.6 mV, respectively, demonstrating a good dispersion. The stability studies indicated that there were no significant changes detected in particle size and particle surface charge of the polymeric nanoparticles after storage at 4 °C for 1 month, which demonstrated the excellent stability of these particles during storage. The drug release kinetics in vitro of the nanoparticles was evaluated at pH 5.5 (simulating the endosome site of the cancer cells) and pH 7.5 (simulating the physiological environment) employing the dynamic dialysis method, and they displayed redox- and pH-sensitive drug release. In comparison with the excellent stability in storage, the drug release mechanism of the polymeric nanoparticles was high at 37 °C during dynamic dialysis, showing that it may be attributed to the temperature. MTT assay was performed on A549 cancer cells to study the cytotoxicity in vitro of the nanoparticles and the free drugs [72]. The dual drug-loaded nanoparticles exhibited high cytotoxicity which was 4-fold effective in cell growth inhibition when compared to the free curcumin on the A549 cancer cells. However, the polymeric nanoparticles caused greater growth inhibitory effect when compared to the individual drug. In vivo studies in A549 xenograft tumor-bearing nude mice revealed that the accumulation of drugs at the tumor site was high when compared to the free drug. The nanoparticles displayed a synergistic effect in vivo which was significant with improved reduced toxicity. After injection of the mice with the formulation at a selected time duration of 1, 6, 12, and 24 h, respectively, the concentrations of the drugs in the tumor site were 1.45, 3.31, 2.61, and 0.93 μg/g, respectively, for the nanoparticles. The drug concentrations were higher for the nanoparticle formulation when compared to the free drug, which were 1.42, 1.25, 0.67 and 0.58 μg/g, respectively [72].

Lotfi-Attari et al. developed PEGylated PLGA for the combination of curcumin and chrysin to treat colorectal cancer [73]. The physicochemical properties of the nanoparticles were successfully studied using Fourier transform infrared spectroscopy (FTIR), DLS, transmission electron microscopy (TEM), and scanning electron microscopy (SEM). The in vitro cytotoxicity studies revealed that the individual drugs and drug-loaded nanocarriers demonstrated dose-dependent cytotoxicity activity against Caco-2 colorectal cancer cells, and specifically, dual drug-loaded polymeric nanoparticles had more synergistic antiproliferative outcome and, importantly, inhibited the growth of colorectal cancer cells when compared to the free drugs [73]. The enhanced anticancer effect of drug-loaded nanoparticles is due to the controlled release of the drugs and high intracellular concentrations when compared to the free drugs that diffuse easily into the cell membrane. Medel et al. prepared methoxy-poly(ethylene glycol)-block-polylactic acid (mPEG-b-PLA)-based nanoparticles encapsulated with curcumin and bortezomib [74]. The particle sizes of the polymeric nanoparticles were in the range of 100–150 nm. The cellular uptake studies revealed a maximum cellular uptake in the MCF-7, HeLa, MDA-MB 231, and HeLa cancer cells after 3 h. The in vitro cytotoxicity studies of polymeric nanoparticles using MTT assay on the three cancer cell lines revealed high growth inhibition when compared to the free drugs [74].

Some nanoparticles of curcumin are currently in a clinical trial. Imx-110 is a combination therapy designed for the inhibition of cancer resistance and the induction of apoptosis. It is composed of NF-kB/Stat3/pan-kinase inhibitor curcumin and doxorubicin for enhanced tumor penetration. The formulation was tailored, resulting in their capability to bind to various targeting moieties, thereby delivering the loaded drugs to the tumors. In 2018, positive interim results were reported from a phase 1b/2a study testing the formulation in advanced solid tumors. No adverse events were reported in patients that enrolled for the studies [75].

When the formulation is administered, the curcumin moiety inhibits STAT3- and NF-kB-mediated signaling pathway targets by hindering the activation of STAT3 and NF-kB. These signaling pathways play a key role in uncontrolled tumor cell proliferation and tumor resistance to mechanisms such as metastasis, apoptosis, etc., which are activated in different human cancers. The doxorubicin moiety acts by intercalating into the DNA resulting in interference with the topoisomerase II activity. The aforementioned mechanisms result in the inhibition of DNA replication and RNA synthesis whereby tumor cell growth is inhibited. The capability of the formulation to inhibit NFkB and STAT3 activity can overcome the mechanisms of multidrug resistance in tumor cell mechanisms, making it effective in chemoresistant tumor cells [76].

NanoCurc^™^ was developed for the treatment of pancreatic and brain cancer. The initial study revealed that it increases the bioavailability of curcumin and inhibits tumor growth in vivo. The combination of the formulation with gemcitabine inhibited tumor growth. The formulation displayed a dose-dependent decrease in selected brain tumor cell lines [77,78,79].

### 4.2. Micelles

**Figure 3:**
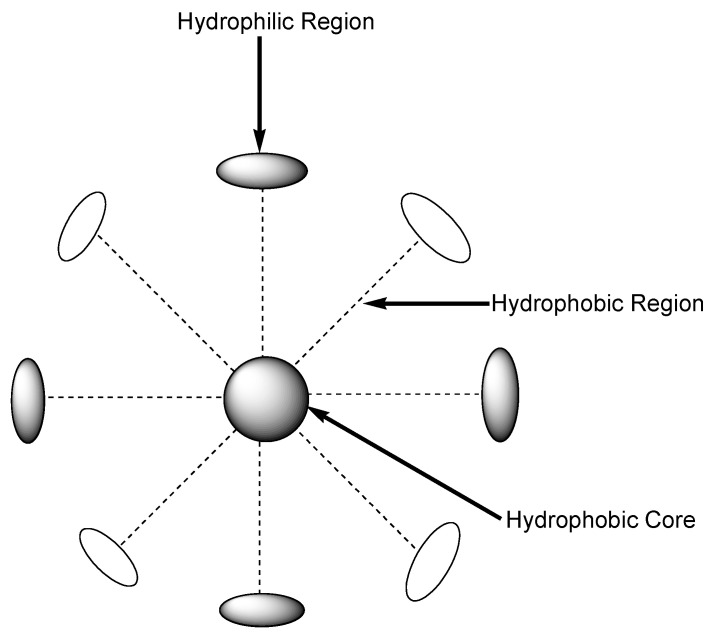
Schematic diagram of micelles.

Micelles are nanocarriers with a particle size that ranges between 10 and 200 nm. They are formed by self-aggregation of surfactant molecules in an aqueous medium (Figure 3) [80]. These drug delivery systems are known by their long hydrophobic chains, which are employed for the entrapment of different therapeutic agents, and a hydrophilic head group [81]. There are several benefits of employing micelles in drug delivery, such as controlled and sustained drug release, enhanced drug pharmacokinetics, increased drug bioavailability, reduced drug toxicity, and improved drug water solubility [82]. Several studies have been reported on the co-delivery of curcumin using nano-sized micelles, as discussed below.

Ma et al. designed hyaluronic acid-vitamin E succinate graft copolymer-based micelles for synergistic co-delivery of doxorubicin and curcumin into tumor cells [83]. The micelles displayed spherical-like morphology with a particle size of 223.8 nm and a negative surface charge of −10 ± 0.12 mV because of carboxyl groups on the surface area of hyaluronic acid. The drug encapsulation efficiency of curcumin and doxorubicin in co-loaded micelles were 72.5% and 94.8%, respectively, with a drug loading efficiency of 8.3%. The drug release profiles of the loaded drugs were sustained release at pH 4.5 (simulating lysosomal pH), 5.5 (simulating endosomal pH), 6.5 (simulating tumor extracellular pH), and 7.4 (simulating physiological pH). At pH 7.4, 27% doxorubicin and 37% curcumin were released from the drug loaded micelles indicating that the micelles’ structure integrity in physiological condition was retained. However, the release of both drugs was rapid at pH 5.5 and pH 4.5 over a period of 24 h. The in vitro cytotoxicity of the co-drug encapsulated micelles on lung cancer cells (MCF-7/Adr) using MTT assay revealed significant cytotoxicity when compared to the free drug solutions of either doxorubicin or doxorubicin-curcumin [83]. Furthermore, in vivo antitumor activity of the co-drug loaded micelles using mice bearing 4T1 tumor (breast cancer) indicated a significant inhibition and reduction of the tumor when compared to animal models treatment with the free drugs on day 12. The tumor inhibition rates were 21.3%, 27.61, 36.05%, 36.47%, and 55.23% for doxorubicin solution, doxorubicin + curcumin, doxorubicin-loaded micelles, doxorubicin-loaded micelles + curcumin, and micelles loaded with both drugs, respectively, revealing the synergistic effects of combining both drugs [83]. Incorporating doxorubicin in combination with curcumin into the micelles reduced the cardiotoxic and hepatoxic effect of doxorubicin revealing the efficacy of combining anticancer drugs with curcumin.

Yang and co-workers prepared poly (ethylene glycol)-benzoic imine-poly(g-benzyl-L-aspartate)-b-poly(1-vinylimidazole) block copolymer (PPBV)-based micelles co-loaded with curcumin and paclitaxel for synergistic therapeutic effect on breast cancer [84]. The average particle size of a drug-loaded polymeric micelles ranged between 42 and 80 nm with a weak negative surface charge of −2.26 ± 3.9 mV and polydispersity index (PDI) of 0.112 ± 0.15. The entrapment efficiency of curcumin and paclitaxel in the micelles was 30.9% and 35.4%, respectively. The in vitro drug release of the drugs paclitaxel and curcumin were 61% and 67% at pH 7.4, 76% and 71.5% at pH 6.5, and 95% and 89% at pH 5.0, respectively. The drug release profiles of the micelle formulation indicate that at pH 7.4, the benzoic-imine bond was stable, hindering the release of the hydrophobic drugs. At pH 5.0 ad 6.5, the PEG layer was detached gradually from the micelles resulting in the rapid release of both drugs. The release profiles of both drugs were influenced by the pH. The in vivo antitumor studies of the dual-loaded micellar system using mice bearing subcutaneous MCF-7 tumors exhibited high inhibition of tumor growth without any significant recurrence when compared to single drug-loaded micelles and free drugs. The average volume of the tumors treated with the co-loaded micelles was 4.7, 5.2, 5.4, 6.6-fold smaller when compared to curcumin-loaded micelles, paclitaxel-loaded micelles, free curcumin-paclitaxel, and 0.9% NaCl (control), respectively. Furthermore, the cytotoxicity efficacy of the polymeric micelles was studied using MCF-7 mammosphere cells in vitro; co-encapsulated PPBV micelles exhibited higher inhibitory potency when compared to free drugs and control [84]. The presence of the pH-responsive linkage, benzonic imine, and pH-sensitive charge-switching segment can promote extended blood circulation, increase cellular uptake, and enhance drug accumulation in the tumor. The co-delivery system loaded with both drugs inhibited the proliferation of both cancer cell lines, non-breast cancer stem cells and breast cancer stem cells.

Scarano et al. formulated dual drug-loaded polymeric micelles for the combination of curcumin and oxoplatin [85]. Triblock copolymer based on the PEG shell, biodegradable polycaprolactone PCL, and an amine bearing polymer as the interphase was used for the incorporation of oxoplatin synthesized by the amalgamation of RAFT polymerization and ring-opening polymerization. Particle size analysis of the dual drug polymeric micelles was 38 nm, which was confirmed using TEM analysis. The encapsulation efficiency of curcumin into the micelles was in the range of 6–7 wt%. The cytotoxicity efficacy of dual drug-loaded micelles, individual micelles, and free drugs was studied on A2780 human ovarian cancer cell lines employing the standard SRB assay. The unloaded micelles exhibited very low growth inhibition of A2780 cancer cells. The in vitro cytotoxicity assessments further showed that the cytotoxic effect of the encapsulated drug increased when compared to the free drugs. In addition, the dual drug-loaded nanomicelles displayed an enhanced synergistic effect, resulting in a combination index of 0.2–0.35 when compared to the curcumin and oxoplatin combined together without nanomicelles, already displaying a synergy with a combination index ranging between 0.4 and 0.8. Incorporating both drugs whereby curcumin was physically encapsulated into the triblock copolymer with a crosslinking using oxoplatin at the interphase enhanced the structural integrity of the micelles thereby promoting a sustained release of curcumin. It also prevented the premature disassociation of the delivery system [85].

One micelles formulation of curcumin is currently in clinical trial. Kocher et al. reported micellar formulation of curcuminoids, which were administered simultaneously with a mixture of the adjuvants, such as xanthohumol, sesamin, ferulic acid, and naringenin, in a single-blind, human study [86]. The micelles formulation was composed of 7% curcuminoid powder and 93% Tween-80. The amount of curcuminoid in the blood samples was analyzed, and the bioavailability of the major curcuminoid was high when compared to the free drug. The formulation was reported to be safe and useful for the prevention of inflammatory diseases.

### 4.3. Nanoliposomes

**Figure 4:**
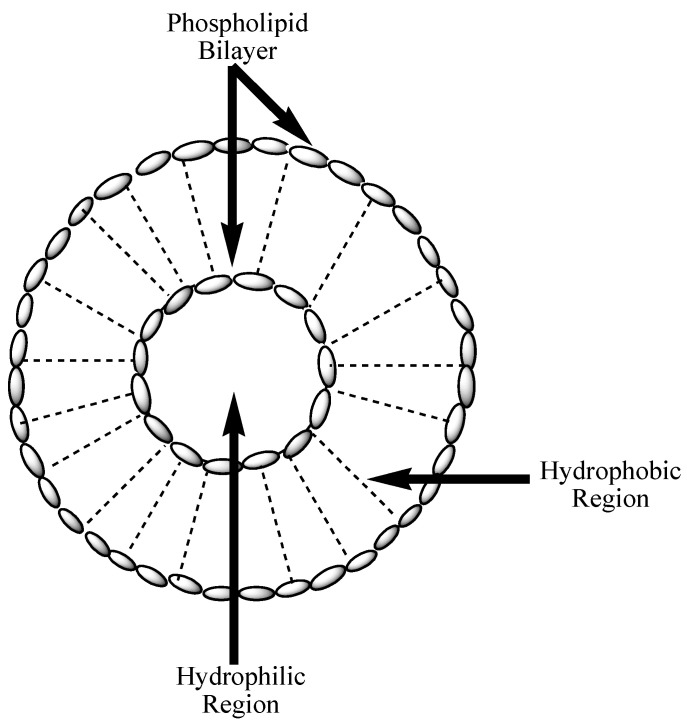
Schematic diagram of nanoliposomes.

Nanoliposomes are artificial and smaller spherical-shaped vesicles formulated from phospholipid and cholesterol (Figure 4) [87]. They exhibit the size range of 30 nm to a few micrometers. These nanocarriers display both hydrophobic and hydrophilic features with good biocompatibility. Their features are different in relation to zeta potential, particle size, the composition of the lipids, and the method of preparation [87]. Nanoliposomes consist of lipid bilayers surrounded by aqueous components, whereby the polar head groups are oriented in the pathway of the interior and exterior aqueous phases. The form of their bilayers, such as bilayer charge, permeability, and rigidity, is influenced by the constituents utilized for their preparation. They are suitable for the delivery of both hydrophilic and hydrophobic therapeutic agents [88]. The advantages of liposomes include low drug toxicity, biocompatibility, biodegradability, etc. They can be utilized for the encapsulation of hydrophilic and lipophilic drugs and they promote site-specific drug delivery [89]. The aforementioned advantages make nanoliposomes very suitable in the co-delivery of chemotherapeutic drugs.

Ruttala and Ko prepared and evaluated PEG-based nanoliposomes for the combination of curcumin and paclitaxel [90]. The DLS experiment revealed an average particle size of 108.21 nm with a PDI of 0.2 and a negative surface charge of 2.65. The encapsulation efficiency (EE) and loading capacity of curcumin were 99.9% and 9.09%, and that of paclitaxel was 99% and 5.9%, respectively. TEM images showed a distinct spherical-shaped morphology. The drug release kinetics of the nanoliposomes in vitro was sustained for three days at pH 7.4. The release of curcumin and paclitaxel was sequential, whereas the release of curcumin was faster when compared to paclitaxel. Almost 35% of the curcumin was released from the nanoliposomes in 24 h, whereas less than 20% of paclitaxel was released in the same period. The in vitro cytotoxicity studies showed that the drug-loaded nanoliposomes displayed superior cytotoxic effect against MCF-7 and B16F10 when compared to combining the free drugs and the individual free drugs [90]. The sustained release profiles of both drugs from the nanoliposomes also contributed to their significant cytotoxic effects in vitro.

Barui and co-workers developed pegylated RGDK-lipopeptide liposomes for the co-delivery of curcumin and doxorubicin to tumor vasculature of cancer cells [91]. The hydrodynamic particle size diameter and zeta potential of the nanoliposomes was in the range of 190–230 nm and 2–4 mV, respectively. The nanoliposomes drug entrapment efficiency for curcumin and doxorubicin was 85% and 100%, respectively. The cellular uptake studies of the nanoliposomes were carried out in HUVEC cancer cell lines and the uptake efficiencies were compromised in HUVEC cells pre-incubated with monoclonal antibodies against αVβ3 and αVβ5 integrin receptors. In vivo studies on C57BL/6J mice bearing the aggressive B16F10 tumors separately administered the liposomal formulations of only curcumin, only doxorubicin, and liposomal formulations containing both drugs, intravenously. The tumor growth inhibition was significant in mice treated with formulations containing both drugs. The tumor growth inhibition was 2–3 folds less in mice treated with formulations containing only a single drug. Mice administered with the control liposomal formulations developed large tumors on day 24. Furthermore, administration of the formulation containing both drugs improved the survival rate of the tumor bearing mice revealing the efficacy of curcumin in alleviating the adverse side effects of doxorubicin. The enhanced accumulation of doxorubicin in the tumor vasculature 24 h after intravenous administration of the liposomal formulation containing both drugs also revealed the efficacy of curcumin in inhibiting doxorubicin efflux from tumor endothelial cells. The in vitro cytotoxicity experiments on nanoliposomes were performed using MTT assay against B16F10 and HUVEC cancer cells. The synergistic cytotoxic effects of liposomes encapsulated with doxorubicin and curcumin destroyed the tumor and tumor endothelial cells [91].

Liposome formulations of curcumin in clinical trials have been reported. Storka et al. reported a phase I clinical trial on the pharmacokinetics and safety of an intravenously administered liposomal formulation of curcumin, Lipocurc™. It was safe up to a dose of 120 mg/m^2^ [92]. Bolger et al. reported the pharmacokinetics of curcumin and plasma levels of tetrahydrocurcumin (THC) in cancer patients and healthy individuals after intravenous infusion of Lipocurc™ formulation. Three drugs that target the renin-angiotensin system, Ramipril, Lisinopril, and Valsartan, increased the plasma levels of curcumin and THC in three cancer patients infused with Lipocurc™. The co-medications influenced the pharmacokinetics of the curcumin infusion formulation in cancer patients [93].

### 4.4. Polymer-Drug Conjugates

**Figure 5:**
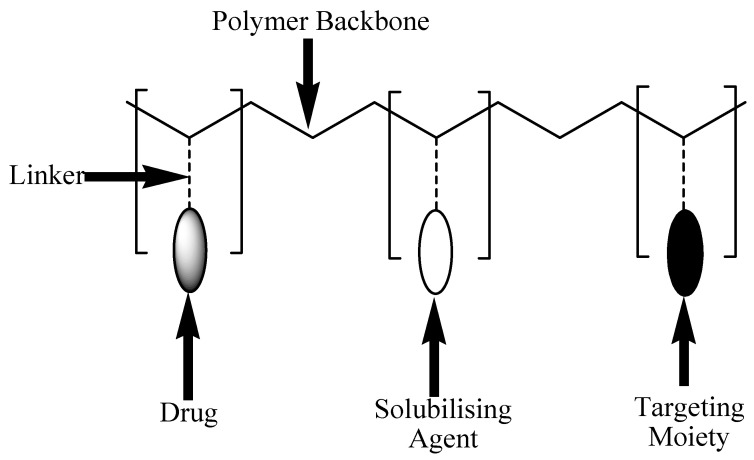
Schematic Diagram of polymer-drug conjugates.

Polymer-drug conjugates which are also known as polymer prodrugs are nanocarriers that are designed to covalently incorporate therapeutic agents into the polymer backbone through selected functional groups (Figure 5) [94,95]. The functional groups that are usually used include alcohol, amines, carboxylic groups, etc. [96]. The concept of these nanocarriers was first proposed by Helmut Ringsdorf in 1975 [94]. Polymer prodrugs are composed of biodegradable polymer backbone, where there are three special units. These three units are (i) a solubilizing unit that influences the water solubility of the nanocarrier soluble, (ii) covalently-linked therapeutic agents, and (iii) a targeting moiety that transports the nanocarriers to the targeted environment [97,98]. Polymer prodrugs can be formulated from linear polymeric carriers such as polyaspartamides, poly(vinyl alcohol), poly(ethylene glycol) (PEG), poly(malic acid), and poly(vinyl pyrrolidone) [99]. They can also be formulated from branched polymeric carriers such as polymeric micelles and poly (amidoamine) (PAMAM) and poly (ethyleneimine) dendrimers [99]. They exhibit several unique advantages such as targeted drug delivery, biocompatibility, non-toxicity, enhanced drug water solubility, improved drug stability, and drug bioavailability [100,101,102].

Dong et al. successfully prepared oligosaccharides of hyaluronan (hydrophilic polysaccharide)-based polymer-drug conjugates incorporated with curcumin and alendronate via disulphide linker [103]. Proton Nuclear Magnetic Resonance Spectroscopy (^1^HNMR) was employed to confirm the physiochemical structure of the conjugates. The average particle size of the conjugates was 179 nm with a negative zeta potential of −25.7 mV. The morphology studied using SEM analysis was a spherical-shape morphology with a particle size of 80 nm. The PI of these nanocarriers was 0.14, which was smaller than 0.2, with a drug loading and % encapsulation efficiency of 6.38% and 52.58%, respectively. The cytotoxicity activity of the dual drug-loaded nanocarriers and the free drug was assessed in vitro against breast cancer cell lines (MCF-7 and MDA-MB-231) utilizing MTT assay. The nanocarriers showed the lowest cell viability after two days when compared to free curcumin. The uptake and cytotoxic effect of the nanocarriers were higher in MDA-MB-231 cells when compared to MCF-7 cells because of a higher expression of the CD44 receptor in the MDA-MB-231 cells. The disulphide bond was a connection arm of the conjugate, and it enhanced the drug loading and encapsulation efficiency of curcumin. It also promoted tumor targeting of the conjugate [103].

Zhang et al. prepared pH-responsive PEG-based conjugates for the combination of curcumin and doxorubicin to treat cancer [104]. DLS and TEM analysis of the co-loaded nanocarrier showed an average hydrodynamic diameter of 183.5 nm with a low negative zeta potential and spherical shape morphology, respectively. The drug loading efficiency of curcumin and doxorubicin was 18.2% and 18.35%, respectively. The in vitro drug release studies showed a rapid release of curcumin and doxorubicin from the conjugates at pH 5.0 in 24 h resulting from the cleavage of the oxime linker. The cellular uptake and internalization of the conjugates were evaluated on HepG 2 cancer cells using fluorescence microscopy. The conjugates were taken up efficiently by HepG 2 cancer cells, which were distributed predominately in the cellular cytoplasm after 2 h of incubation. The fluorescence images acquired after 4 h of incubation displayed high fluorescence intensity of the incorporated drugs within the cells. Furthermore, in vitro cytotoxicity studies showed that the dual drug-loaded polymeric conjugates displayed a higher anti-tumor efficacy against HepG 2 and HeLa cancer cells when compared to the free drug and conjugates incorporated with one drug. Improved anti-tumor efficacy of the conjugates was also observed in vivo signifying good tumor inhibition efficacy of the conjugates [104].

Hong et al. prepared U11 peptide decorated pH-sensitive polymer prodrugs for the combination of curcumin and doxorubicin to combat lung cancer [105]. ^1^HNMR confirmed the successful incorporation of the drugs via amido linkage. The TEM analysis of the nanocarriers displayed spherical shape morphology with a core-shell structure. The average particle size and surface charge of the conjugates using Zetasizer Nano was 121.3 nm and −33.5 mV, with a curcumin and doxorubicin encapsulation efficiency of 90.5 and 81.7%, respectively. The drug release studies of the nanocarriers were performed in vitro at pH 7.4, 6.0, and 5.0. The release of both drugs was pH-dependent, and the release rate increased with the decreasing pH value. U11 peptide decorated nanocarriers displayed significantly higher cellular uptake efficiency when compared to the non-U11 peptide decorated nanocarriers. The dual drug loaded conjugates showed better cytotoxicity in vitro against A549 lung cancer cells when compared to the free drugs. The drug-loaded conjugates and the free drugs displayed almost the same cytotoxicity on HUMEC cancer cell lines. Decorated nanocarriers showed improved inhibition of the tumor growth in vivo when compared to the non-decorated nanocarriers and free drugs. After 1 h of administering the drug-loaded nanoparticles, the drug in the heart and kidney was low when compared to the free drugs, which were significantly high. The sustained release mechanism of the nanoparticle formulation resulted in reduced systematic toxicity with decreased systemic toxicity and improved anti-tumor activity [105].

### 4.5. Dendrimers

**Figure 6:**
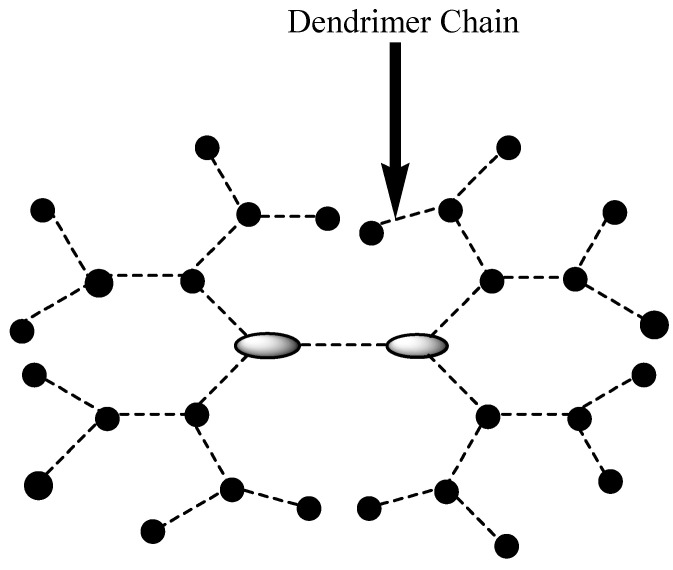
Schematic diagram of a dendrimer.

Dendrimers are drug delivery systems that are monodispersed and highly branched with a three-dimensional structure and spherical globe shape (Figure 6) [106]. These polymeric nanocarriers are very useful in drug delivery because of their low polydispersity index, enhanced biocompatibility, and controlled molecular weight [107]. They have functional groups at the terminal layers that are appropriate for the entrapment of drugs and the combination of targeting moiety [107]. Their intramolecular cavity is important for the encapsulation of drugs for a controlled and sustained drug release mechanism, improved drug delivery, reduced drug toxicity, and improved drug efficacy [108].

Ghaffari and co-workers developed polyamidoamine (PAMAM)-based dendrimers for the co-delivery of curcumin and Bcl-2 siRNA against HeLa cancer cells [109]. The particle size analysis of co-encapsulated polymeric dendrimers was 180 nm with a zeta potential of −48 mV. The drug release was performed in vitro at pH 5.4 (simulating tumor environment) and 7.4 (simulating physiological conditions). Less than 5% of curcumin was released over a period of 10 h from the polymeric dendrimers at both pH values, followed by a sustained release at pH 5.4 with 40% release of curcumin in three days. At pH 7.4, only 10% of curcumin was released in seven days. The in vitro cellular uptake studies of co-loaded dendrimers into HeLa cells using fluorescent microscopy showed higher internalization of the drugs from PAMAM dendrimers. The dendrimers co-loaded with curcumin and Bcl-2 siRNA exhibited in vitro HeLa cell growth inhibition in a time- and concentration-dependent manner. The co-delivery of curcumin and Bcl-2 siRNA resulted in enhanced cellular drug uptake [109].

### 4.6. Hydrogels

**Figure 7:**
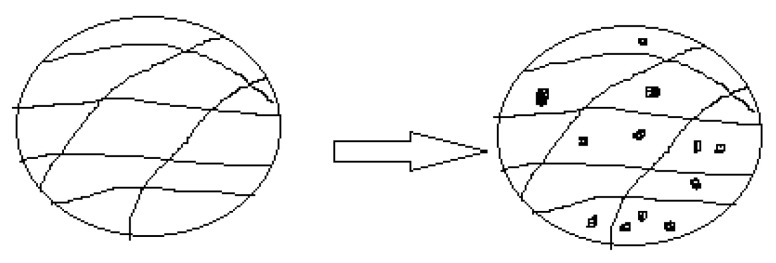
Schematic diagram of a hydrogel.

Hydrogels are polymeric carriers with a three-dimensional network (Figure 7). They are prepared from synthetic polymers, biopolymers, or the combination of both classes of polymers [110]. These systems can absorb and retain a large amount of water and biological fluid. There are several features that influence the degree of porosity of hydrogels such as the polymer composition, method of preparation, and the nature of materials used to prepare them. They can be tailored into different forms such as films, slaps, microparticles, and nanoparticles [111]. The unique properties that are displayed by hydrogels include their non-toxicity, good biocompatibility, non-immunogenicity, affordability, patient compliance, controlled drug release rate, etc. [110]. They are also very useful in combination therapy. All the aforementioned properties make hydrogels very useful in biomedical applications such as diagnostics, regenerative medicine, tissue engineering, separation of biomolecules, cellular immobilization, drug delivery, etc. [37]. Hydrogels have been studied extensively for the treatment and management of chronic diseases such as malaria, cancer, tuberculosis, human immune virus (HIV), and influenza. They can be administered via parenteral, nasal, oral, pulmonary, and nasal routes, etc. [112].

Pushpalatha et al. formulated a cyclodextrin nanosponge-based hydrogel for transdermal co-delivery of curcumin and resveratrol for treatment of breast cancer [113]. Curcumin and resveratrol content in the hydrogels demonstrated a high loading efficiency of the nanocarriers. TEM analysis displayed a porous-like morphology of the co-loaded hydrogels. Curcumin-loaded and resveratrol-loaded hydrogel displayed a dose-dependent effect on the MCF7 breast cancer cell with an IC_50_ value of 10.11 μg/mL and 26.19 μg/mL, respectively. A combination of curcumin-loaded and resveratrol-loaded hydrogel demonstrated a significant cytotoxic effect in a concentration-dependent mode (*p* < 0.05) with an IC_50_ value of 3.41 μg/mL when compared to the single treatment of curcumin-loaded and resveratrol-loaded hydrogel. The nanoformulations exhibited a cytotoxic synergistic effect on the MCF-7 breast cancer cells with a combination index value of 0.29. An ex vivo skin permeation study revealed an 11.5 fold and 2.4 fold permeation enhancement of curcumin and resveratrol, respectively. The combination of curcumin and resveratrol nanosponges results in a high cytotoxic effect against breast cancer cells suggesting that the nanosponges are an effective nanocarrier for transdermal delivery of curcumin and resveratrol [113].

### 4.7. Nanocapsules

**Figure 8:**
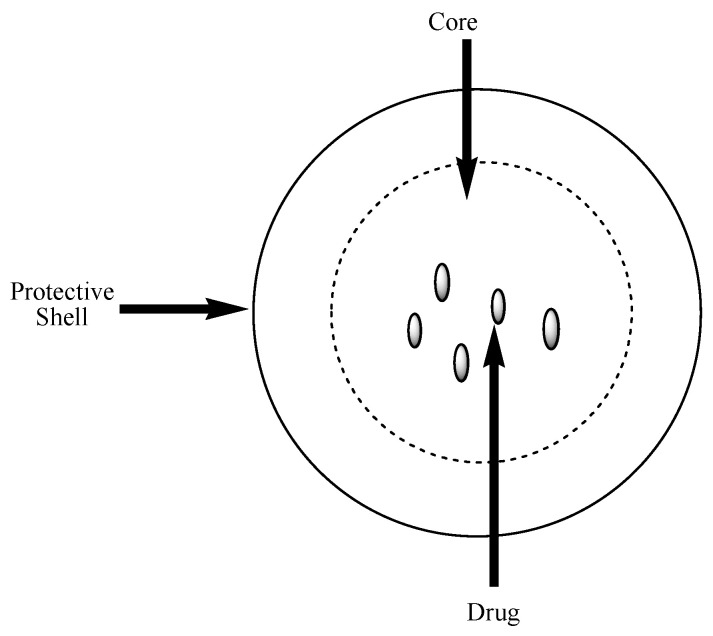
Schematic diagram of nanocapsules.

Polymer-based nanocapsules are nanocarriers that are composed of a core and protective shell where the drugs are encapsulated (Figure 8) [114]. Various methods are employed to prepare polymeric capsules such as monoemulsion polymerization, self-assembly of block copolymers, solidification of droplet shell, etc. [115]. The particle size of nanocapsules is usually in the range of 10–1000 nm [116]. There are several advantages of polymeric nanocapsules in drug delivery such as high drug-encapsulation and loading efficiency, reduced drug toxicity, sustained and controlled drug delivery, improved drug biodegradability, and bioavailability [117].

Slika et al. formulated polymer-based nanocapsules via the self-assembled nanoprecipitation method encapsulated with curcumin and piperine, using poly (allyl amine) hydrochloride as the carrier [118]. The physiochemical properties of the polymeric nanocapsules were evaluated using FTIR, XRD, SEM, DLS, and zeta potential analysis. The FTIR, SEM images, and XRD spectrums exhibited the expected functional groups, spherical shaped morphology, and the amorphous nature of the polymers, confirming successful encapsulation of the drugs into the capsules. Particle size analysis using DLS showed the particle size diameter of the nanocarriers was in the range of 80–100 nm, while zeta potential analysis displayed +27 mV. The encapsulation efficiency of curcumin in nanocapsules ranged between 83.1% and 96.2%, depending on the curcumin ratios per nanocapsule. The drug release studies in vitro at 37 °C displayed that pH generated the maximum curcumin release in basic conditions when compared to neutral and acidic medium and followed the Higuchi model. In vivo and in vitro cytotoxicity studies exhibited that drug-loaded polymeric nanocapsules exerted potential and selective cytotoxic activity against Caco-2-cancer cell lines. Furthermore, curcumin-loaded nanocarriers improved the cytotoxic efficacy of curcumin by 3-fold [118].

### 4.8. Exosomes

**Figure 9:**
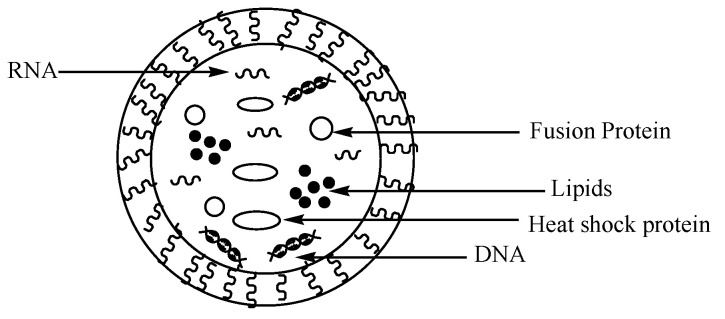
A schematic diagram of exosomes.

Exosomes are classified as extracellular vesicles which are obtained from plants and animals (Figure 9). They have been reported as a potential nano-based delivery system for bioactive agents due to their distinct properties such as their safety, good biocompatibility, excellent stability, small size, biorecognition, bioadhesive nature, and capability to release active agents at the target site. Their size is in the range of 30–100 nm [119]. They are composed of lipids, proteins, etc. which are randomly accumulated from the parent cell. Their phospholipid bilayer exterior is made up of cell surface proteins which promote their intercellular communication, immune surveillance, etc. [120]. They are composed of different types of lipids, such as saturated fatty acid chains, ceramides, phosphoglycerides, cholesterol, and sphingolipids [121]. Their composition is crucial, and they act as biomarkers. Their composition also influences their functions in biological processes.

Bioactive agents are loaded into exosomes by two known major approaches, which are active and passive drug encapsulation approaches. The loading efficiencies and stabilities of the drugs in the exosome vesicles using both approaches differ [122]. The active drug encapsulation approach includes extrusion, freeze and thaw cycles, incubation with membrane permeabilizers, sonication, electroporation, and the click chemistry method. In the active drug loading approach, the membrane integrity of the exosomes is compromised resulting in the diffusion of the drug into the exosomes. The drugs are encapsulated inside the exosomes and also attached on the outer layer of the membrane, resulting in two phases of drug release [123]. In the click chemistry method, small molecules and macromolecules are attached to the surfaces of exosomes through covalent bonds. Copper-catalyzed azide alkyne cycloaddition, known as click chemistry, is ideal for bioconjugation of small molecules and macromolecules to the surfaces of exosomes [124]. This drug conjugation approach is mild and does not affect the function, size, and structure of the exosomes.

In the passive drug loading approach, the exosomes are incubated with the drug and the drug loading efficiency is influenced by the nature of the drugs. However, this approach is limited by low drug loading capability [125,126]. Due to the unique features of exosomes, they have been designed for the delivery of curcumin, resulting in good therapeutic outcomes in vivo. Researchers have reported the design of exosomes for the delivery of curcumin. However, there is only one report on the design of exosomes for co-delivery of curcumin and other anticancer agents. Jia et al. conjugated neuropilin-1-targeted peptide with the membrane of exosomes loaded with a combination of curcumin and superparamagnetic iron oxide nanoparticles via the click chemistry method. The exosome formulation efficacy was studied on glioma cells. In vivo studies were performed by administering the formulation on orthotopic glioma models. The capability of the exosomes to cross the Blood Brain Barrier (BBB) indicated that the formulation is suitable for targeted imaging of glioma. Furthermore, the combination of the nanoparticles with curcumin resulted in a significant synergistic anticancer effect. These findings revealed that exosomes can be employed for the diagnosis and treatment of brain cancer [127].

Researchers have reported the efficacy of loading curcumin into exosomes. Sun et al. reported curcumin complexed with exosomes. The exosomes induced apoptosis when taken up in the activated monocyte-derived myeloid cells in the peripheral blood. They also improved the bioavailability and stability of curcumin in vivo. A post-intraperitoneal injection of curcumin-loaded exosomes in mice revealed the accumulation of the formulation in the liver, lungs, and spleen [126]. Exosomes isolated from raw bovine milk were loaded with curcumin [128]. In vitro studies on different cancer cell lines showed that growth inhibition of the curcumin-loaded exosomes was 66% against H1299 lung cancer cells and 61% against A549 lung cancer cells, when compared to free curcumin, which was 23% and 34%, respectively, at 1.56 μM concentration. The growth inhibition effect of drug loaded exosomes against cervical and breast cancer was significant with 70% and 80%, when compared to the free curcumin, which was 10% and 35%, respectively. In vivo studies were performed using oral gavage of exosomes or drug loaded exosomes at (20 mg/kg b. wt.) three times a week. A high inhibition of tumor growth of 61% was observed in animal models treated with the drug-loaded exosomes, when compared to treatment with exosomes alone, which was 21% [128]. The clinical trial of curcumin conjugated with exosomes to investigate its delivery of curcumin to colon tumors is ongoing. [129].

## 5. Conclusions

In this review article, polymer-based nanocarriers co-loaded with curcumin and selected anticancer drugs were reported for the treatment and management of various types of cancer have been described. The incorporation of the drugs into the polymeric nanocarriers resulted in reduced drug toxicity, overcame drug resistance, influenced the drug release mechanism of the drugs from the systems by exhibiting controlled or sustained drug release profile, improved the water solubility of loaded drugs, and were suitable for combination therapy. Most of the nanocarriers in which anticancer drugs were co-loaded with curcumin resulted in a synergistic cytotoxic effect, which has the potential to overcome drug resistance. Although polymeric nanocarriers offer several advantages, there are some limitations that need to be thoroughly investigated by researchers, such as their toxicological profiles over a long term biomedical application, high cost, low drug loading/encapsulation, unclear mechanism of action against cancer cells, etc. There is also a need for most of the studies currently reported to reach clinical trial phase. However, with the number of research reports currently reported in the field of development of nanocarriers as drug delivery systems, there is no doubt that in the next few years from now, most of these systems will be readily available for clinical use with potent therapeutic outcomes.

## Figures and Tables

**Table 1:** Efficacy of curcumin in clinical trials.

Types of Cancer	Drugs Administration	Outcomes	References
Metastatic colorectal cancer	2 g of curcumin plus folinic acid/fluorouracil/oxaliplatin	CXCL1 was not altered over time significantly. The combination was safe and tolerable for the patients	[43]
Pancreatic cancer	Combination of curcumin 8 g with gemcitabine-based chemotherapy.	The median survival time after the administration of curcumin was 161 days with 1-year survival rate. The plasma curcumin levels in 5 patients were in the range of 29 to 412 ng/mL. The combination was found to be was safe.	[44]
Metastatic breast cancer	Combination of 100 mg per day of docetaxel intravenously and curcumin 6000 mg per day orally	The combination was safe and tolerable.	[45]
Chronic myeloid leukemia	Combination of turmeric powder with imatinib	The levels of nitric oxide were significantly decreased.	[46]
Chemotherapy-naive metastatic castration-resistant prostate cancer	Combination of docetaxel with curcumin 6000 mg/d.	The combination did not result in any adverse effect and it was well tolerated by the patients.	[47]
Pancreatic cancer	Combination of curcumin formulation (Meriva^®^) with gemcitabine.	The median progression free survival and overall survival were 8.4 and 10.2 months, respectively. The combination was safe and can translate into a good response rate in first line therapy of advanced pancreatic cancer.	[48]
Metastatic colorectal cancer	MB-6 (composed of fermented soybean extract, green tea extract, Antrodia camphorata mycelia, spirulina, grape seed extract, and curcumin extract) with leucovorin/5-fluorouracil/oxaliplatin	The patients displayed a significant lower rate of disease progression and incidence of adverse events.	[49]
Solid tumors	Curcuminoids 180 mg/day in combination with 5-fluoracil–based chemotherapy.	The combination resulted in a significant improvement in health-related quality of life when compared to the placebo (*p* < 0.001). The magnitude of reductions in the mediators implicated in systemic inflammation were significant.	[50]
Radiation dermatitis in breast cancer	6 g of curcumin per day plus radiotherapy	A reduced severity of radiation dermatitis in breast cancer patients.	[51]
Non-small cell lung cancer	Curcumin formulation (CURCUViva^TM^) at 80 mg/1 capsule daily in combination with tyrosane kinase inhibitors (TKIs), (gefitinib and erlotinib)	The short-term use of curcumin in combination with EGFR-positive patients treated with TKIs was safe	[52]
Pancreatic or biliary tract cancer	Curcumin formulation (400 mg), (Theracurmin^®^) combination with gemcitabine-based chemotherapy.	No adverse effects were observed in all the patients. The repetitive administration of a high dosage of curcumin did not increase the incidence of adverse effects in cancer patients receiving curcumin together with the gemcitabine-based chemotherapy	[53]
Squamous cell carcinoma cancer	Curcumin	It inhibits IkB kinase b (IKKb) activity in the saliva of head and neck squamous cell carcinoma cancer patients thereby suppressing the expression of proinflammatory cytokines.	[54]
Prostate cancer	Curcumin and isoflavones	The combination reduced the level of prostate-specific antigen (PSA).	[55]
Colorectal cancer	Curcumin	Increased body weight and reduced serum TNF-alpha levels, increased apoptotic tumor cells, and enhanced expression of p53 molecule in tumor tissue. Improved general health of patients.	[56]
Cervical cancer	Curcumin cream formulation (Basant™ containing curcumin extract, amla, aloe vera, reetha) administered via intravaginal route	The HPV clearance rate in Basant cream was significant with (87.7%) with no serious adverse effects.	[57]

**Table 2:** A summary of nanocarriers loaded with a combination of curcumin and selected anticancer drugs.

Nanocarrier	Polymers Used	Drugs Loaded/Incorporated	Cancer Cell Lines Used	Therapeutic Outcomes	Route of Administration In Vivo	References
Nanoparticle	poly(β-amino ester)	Curcumin and doxorubicin	umbilical vein endothelial cancer cell lines (HUVECs) and human liver cancer cell lines (SMMC 7721)	The high cytotoxicity effect of co-encapsulation of doxorubicin and curcumin in polymeric nanoparticles and improved cellular uptake.	-	[68]
Nanoparticle	PLGA	Curcumin and docetaxel	Human prostate carcinoma cells (PC3 cells).	In vivo studies using mice-bearing PC3 tumor xenografts showed that the hybrid nanoparticles inhibited tumor growth without causing any severe side effects	Subcutaneous administration	[69]
Nanoparticles	PLGA	Curcumin and cisplatin	Cervical cancer	The accumulation of the drug-loaded formulation was enhanced in the tumor tissue when compared to the free drugs.	Subcutaneous administration	[70]
Nanoparticles	Poly (ethylene glycol) (PEG)-lipid bilayer coated mesoporous silica nanoparticles	Curcumin and paclitaxel	Breast cancer cell lines	Controlled and sustained drug release profiles from the nanoparticles.	-	[71]
Nanoparticles	mPEG-PLGA	Curcumin and cisplatin	A549 cancer cells	The nanoparticles exhibited high cytotoxicity which was 4-fold effective in cell growth inhibition when compared to the free curcumin on the A549 cancer cells	-	[72]
Nanoparticles	PEGylated PLGA	Curcumin and chrysin	Caco-2 Colorectal cancer	A significant synergistic antiproliferative outcome when compared to the free drugs	-	[73]
Nanoparticles	methoxy-poly(ethylene glycol)-block-polylactic acid (mPEG-b-PLA)-b c	Curcumin and bortezomib	MCF-7, HeLa, and MDA-MB 231 cancer cells	Significant cytotoxic effects of the nanoparticles loaded with drugs when compared to the free drugs	-	[74]
Micelles	Hyaluronic acid-vitamin E succinate graft copolymer	Doxorubicin and curcumin	Lung and breast cancer	Reduced the cardiotoxic and hepatoxic effect of doxorubicin. Enhanced the cytotoxic effects of the drug loaded micelles	Intravenous administration	[83]
Micelles	poly(ethylene glycol)-benzoic imine-poly(g-benzyl-L-aspartate)-b-poly(1-vinylimidazole) block copolymer	curcumin and paclitaxel	Breast cancer MCF-7	Inhibition of tumor growth without any significant recurrence	Subcutaneous administration	[84]
Micelles	PEG and polycaprolactone	Curcumin and oxoplatin	A2780 human ovarian cancer cell lines	Cytotoxic effect of the encapsulated drug increased when compared to the free drugs	-	[85]
Nanoliposomes	PEG	Curcumin and paclitaxel	MCF-7 and B16F10	Sustained drug release profile and high cytotoxic effect of the drug-loaded nanoliposomes in vitro	-	[90]
Liposomes	Pegylated RGDK-lipopeptide	Curcumin and doxorubicin	B16F10 tumors and HUVEC cancer cell line	The survival rate of the tumor bearing mice was high revealing the efficacy of curcumin in alleviating the adverse side effects of doxorubicin. Enhanced accumulation of doxorubicin in the tumor vasculature.	Intravenously	[91]
Polymer-drug conjugates	Hyaluronan	Curcumin and alendronate	Breast cancer cell lines (MCF-7 and MDA-MB-231)	The uptake and cytotoxic effect of the nanocarriers were higher in MDA-MB-231 cells when compared to MCF-7 cells	-	[103]
Polymer-drug conjugates	PEG	Curcumin and doxorubicin	HepG 2 cancer cells	The dual drug-loaded polymeric conjugates displayed a higher anti-tumor efficacy against HepG 2 and HeLa cancer cells when compared to the free drug.	-	[104]
Polymer-drug conjugates	poly(L-histidine) and U11 peptide	Curcumin and doxorubicin	A549 Lung cancer	Decorated nanocarriers showed improved inhibition of the tumor growth in vivo.	Subcutaneous administration	[105]
Dendrimers	Polyamidoamine	Curcumin and siRNA	HeLa cancer cell	The in vitro cellular uptake of the co-loaded dendrimers into HeLa cells was high.	-	[109]
Hydrogel	Cyclodextrin	Curcumin and resveratrol	MCF-7 Breast cancer	A cytotoxic synergistic effect on the MCF-7 breast cancer cells with a combination index value of 0.29		[113]
Nanocapsules	Poly (allyl amine) hydrochloride	Curcumin and piperine	Caco-2-cancer cell lines	Significant cytotoxic effects.	Intraperitoneal administration	[118]
